# Role of Vaccines in Controlling the Spread of COVID-19: A Fractional-Order Model

**DOI:** 10.3390/vaccines11010145

**Published:** 2023-01-09

**Authors:** Isa Abdullahi Baba, Usa Wannasingha Humphries, Fathalla A. Rihan

**Affiliations:** 1 Department of Mathematics, Bayero University, Kano 700006, Nigeria; 2 Department of Mathematics, Faculty of Science, King Mongkuts University of Science and Technology Thonburi (KMUTT), Bangkok 10140, Thailand; 3 Department of Mathematical Sciences, College of Science, UAE University, Al Ain 15551, United Arab Emirates; 4 Department of Mathematics, Faculty of Science, Helwan University, Cairo 11795, Egypt

**Keywords:** COVID-19, fractional calculus, vaccine, mathematical model, stability analysis, existence and uniqueness

## Abstract

In this paper, we present a fractional-order mathematical model in the Caputo sense to investigate the significance of vaccines in controlling COVID-19. The Banach contraction mapping principle is used to prove the existence and uniqueness of the solution. Based on the magnitude of the basic reproduction number, we show that the model consists of two equilibrium solutions that are stable. The disease-free and endemic equilibrium points are locally stably when $R_{0}<1\;$and $R_{0}>1\;$ respectively. We perform numerical simulations, with the significance of the vaccine clearly shown. The changes that occur due to the variation of the fractional order $\left(\alpha \right)$ are also shown. The model has been validated by fitting it to four months of real COVID-19 infection data in Thailand. Predictions for a longer period are provided by the model, which provides a good fit for the data.

## 1. Introduction

Coronaviruses were first identified as zoonotic pathogens in the mid-1960s. Humans, animals, mammals, and birds can be infected with these viruses. Since 2002, severe acute respiratory syndrome (SARS-CoV) and Middle East respiratory syndrome (MERS-CoV) invaded human population [1]. In December 2019, three patients with pneumonia in Wuhan, China, were infected with a virus genetically related to SARS-CoV. As a result, the disease has become a global pandemic. It has spread to 150 countries, infected many people, and killed many of these people. In fact, the COVID-19 data from various countries reveal a variety of prevention methods, including lockdowns, social distancing, early detection of infections, vaccines, and contact tracing.

COVID-19 is believed to spread between people through direct contact, indirect contact (through contaminated objects or surfaces), or close contact with infected people. Among them are saliva, respiratory secretions, and secretion droplets. They are released from the mouth or nose when an infected person coughs, sneezes, speaks, or sings, for example. The infectious droplets from an infected person can enter the mouth, nose, or eyes of anyone within 1 m of the infected person [2,3,4,5]. Infected patients show symptoms similar to influenza (such as breathlessness, sore throat, and fatigue), and are isolated and treated. Respiratory symptoms, fever, cough, shortness of breath, and breathing difficulties are the most common signs of infection. Sometimes the infection causes SARS, kidney failure, pneumonia, and subsequently death. While most cases of COVID-19 infection are mild, in elderly patients and those with cardiac and respiratory disorders, it can lead to pneumonia, acute respiratory distress syndrome, and multi-organ failure. Asymptomatic individuals have strong immunity or herd immunity [6].

Modeling the impact of vaccination can be stretched back many decades [7]. The transmission dynamics of disease in the presence of vaccination changes in a given population can be studied using compartmental or network models. In compartmental models, the infection process is assumed to be homogeneous. Individuals move between these compartments at certain rates governed by parameters that are related to the transmission. On the other hand, network models deal with interactions between individuals or nodes that exist in a given population characterized by certain compositions [8,9]. Depending on how connected individuals are to each other and their position in these networks, their immune type or level changes. For COVID-19, vaccine doses and individuals’ histories of infection clearly influence the power, width, and immunity length. Many people are afraid of receiving vaccines for reasons that they know best [8]. In order to curb the COVID-19 pandemic, vaccination and restrictive measures are required.

In the literature, COVID-19 infections have been studied by many researchers. Many of these researchers have developed models to observe the effect of vaccines on infectious diseases. There are mathematical models for both classical and fractional orders in the literature. A detailed mathematical model was developed and discussed for early infection of COVID-19 in China [9,10,11]. Optimal control analysis of COVID-19 was conducted in Pakistan using real data. Through isolation and quarantine, mathematical modeling is used to study the most effective ways to reduce COVID-19 transmissions. A lockdown has been studied as an effective method of preventing the spread of the disease [12]. An SEIR model was validated and disease control scenarios were studied using real data from Italy and France [13]. Models based on Saudi Arabia’s real data have been developed [14].

Recently, mathematicians have considered fractional-order models to study epidemic diseases. This relates to the precision of the fractional order that supersedes the integer order, because of its ability to accommodate changes instantly and its nonlocal behavior [15,16,17,18]. Caputo and Rieman–Liouville fractional derivatives are the most widely used fractional operators [19,20,21,22], this is because of their similarities and simplicity of use, as discussed in [23,24]. In recent decades, many fields, including biology, engineering, applied mathematics, and economics, have used fractional-order models. Fractional-order differential equations have the advantage of including memory and hereditary characteristics, which are not possible with integer-order equations.

In order to control and prevent COVID-19 spread, it is crucial to understand the dynamics of the people in the vaccinated compartment. We need mathematical models to better understand the parameters associated with vaccination coverage increases. In this regard, fractional-order models are crucial since they take memory into account. Our goal in this paper is to examine the dynamics of COVID-19 transmission using the fractional-order model in the Caputosense and to examine the significance of the vaccine in curtailing the spread of the disease. Many people who dislike vaccines can be made aware of this. Moreover, it will guide public health officials in increasing vaccination coverage.

The paper is organized as follows. Section 2 provides some preliminary definitions and theorems. The formulation of the model is provided in Section 3. Section 4 examines the existence and uniqueness of the model’s solution. Section 5 outlines local stability analyses and derives basic reproduction ratios. Section 6 provides some numerical simulations, followed by discussion and a conclusion in Section 7.

## 2. Preliminary Definitions and Theorems

We provide some theorems and definitions necessary for the following analysis.

**Definition** **1**  ([25])**.** *A gamma function of p>0 is defined as:*

$$\Gamma \left(p\right)=\int_{0}^{\infty }x^{p-1}e^{-x}dx$$

**Definition** **2**  ([25])**.** *The Caputo fractional derivative of order $\alpha \in \left(n-1,\;n\right]$ of $f\left(x\right)$ is defined as:*

DaCxαfx=1Γn−α∫axx−tn−α−1fntdt, n=α+1

*For *α ∈ (0, 1],* the fractional order is defined in the Caputo sense (1), so that introducing a convolution integral with a power-law memory kernel is useful to describe memory effects in dynamical systems.*

**Definition** **3**  ([25])**.** *(Linearity of fractional derivative) Let* f, g  *be continuous and* b, c *be scalars, then:*

DaRLxαbfx+dgx=bDaRLxαfx+dDaRLxαgx

DaCxαbfx+dgx=bDaCxαfx+dDaCxαgx

**Definition** **4**  ([25])**.** *(Contraction)*

*An operator*f:X →X *that maps a metric space onto itself is said to be contractive if for*0<q<1:

$$d\left(f\left(x\right),\;f\left(y\right)\right)=qd\left(x,\;y\right),\;\forall \;x,\;y\in X.$$

**Theorem** **1**  ([26])**.** *(Picard–Banach fixed point)*

*Any contractive operator that maps a metric space onto itself has a unique fixed point. Furthermore, if *f:X →X *is a contractive operator that maps a metric space onto itself and* a *is its fixed point:* $f\left(a\right)=a;$ *then, for any iterative sequence:*

$$x_{0},\;x_{1}=f\left(x_{0}\right),\;x_{2}=f\left(x_{1}\right),\;\ldots ,\;x_{n+1}=f\left(x_{n}\right),\;\ldots$$

*Converges to* a.

*In other words,* a  *is a solution or an equilibrium for a continuous dynamical system and a fixed point for a discrete dynamical system.*

**Theorem** **2**  ([26])**.** *The equilibriums solutions $x^{*}$ of the system $\left(*\right)$ is locally asymptotically stable if all the eigenvalues $\lambda_{i}$ of the Jacobian matrix $\frac{\partial f}{\partial x_{i}}$ evaluated at the equilibrium points satisfy:*

$$\left|arg\left(\lambda_{i}\right)\right|>\frac{\alpha \pi }{2}\;0<\alpha <1$$

**Theorem** **3**  ([26])**.** *Let $x\left(t\right)\in {\mathbb{R}}^{+}$ be a continuous and derivable function. Then, for any time instant $t\geq t_{0}$ and $\alpha \in \left(0,\;1\right):$*

D0Ctα[xt−x∗−x∗lnxtx∗]≤1−xtx∗D0Ctαxt, x∗∈ℝ+

## 3. Model Formulation

Consider a homogeneous mixing within the population, i.e., individuals in the population have equal probability of contact with each other. Using a deterministic compartmental modeling approach to describe the disease transmission dynamics, at any time *t*, the total population $N\left(t\right)=S_{N}+S_{V}+E+I+H+R$ is subdivided into several epidemiological states:

- Susceptible non-vaccinated compartment $(S_{N}):\;\Lambda$ is the rate at which new individuals enter the population through $S_{N}$. People leave this population through natural death µ, or by being vaccinated at the rate θ, or by being infected with COVID-19 at the rate $\alpha_{1}.$
- Susceptible vaccinated compartment ($S_{V}):$ People from $S_{N}$ come into this population at the rate θ. They leave through natural death at the rate µ, or through contact with the infected people at the rate $\alpha_{2}$, or by recovery at the rate ϕ.
- Exposed compartment (E): People come into this population either from $S_{N}$ or from $S_{V}$ at the rates $\alpha_{1}\;and\;\alpha_{2}$, respectively. They leave either through natural death at the rate µ or by progressing to the infectious compartment at the rate β.
- Infectious compartment (I): People come into this compartment from E at the rate β. They leave to the hospitalized compartment at the rate *η*, to the recovered compartment at the rate *Υ*, or through natural and COVID–19 induced death at the rates µ and $d_{1}$, respectively.
- Hospitalized compartment (H): People come into this compartment from I at the rate *η*. They leave to the recovery compartment at the rate π, or through natural and COVID–19 induced death at the rates µ and $d_{2}$, respectively.
- Recovered compartment (R): People come into this class through H at the rate π, through $S_{V}$ at the rate ϕ, or through *I* at the rate *Υ*. They leave the compartment through natural death at the rate µ.

**Figure 1:**
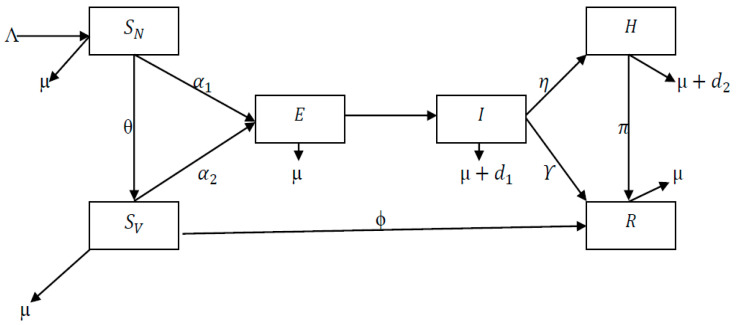
Schematic diagram showing the dynamics of the model.

Figure 1 gives the schematic diagram of the dynamics of the disease. Equation (1) gives the system of the fractional-order model in the Caputo sense as involved in the model.

The transmission dynamics can be described by a nonlinear system of fractional-order differential equations (FODE) of the form:

(1) D0CtαSNt=Λ−α1SNI−θ+µSN,

(2) D0CtαSVt=θSN−α2SVI−ϕ+µSV,

(3) D0CtαEt=α1SNI+α2SVI−β+µE,

(4) D0CtαIt=βE−η+γ+d1+µI,

(5) D0CtαHt=ηI−π+d2+µH,

(6) D0CtαRt=γI+ϕSV+πH−µR. 

$$S_{N}\left(0\right)>0,\;S_{V}\left(0\right)\geq 0,\;E\left(0\right)\geq 0,\;I\left(0\right)\geq 0,H\left(0\right)\geq 0,R\left(0\right)\geq 0.$$

The fractional order gains the model a greater degree of freedom and consistency with the reality of the interactions due to its ability to provide an exact description of the nonlinear phenomena. We next study the existence of positive solutions of such model.

## 4. Existence and Uniqueness of the Solutions

First, we consider the following theorem to show Lipschitz continuity.

**Theorem4.**  *The kernels of Equations (1)–(6) satisfy Lipschitz continuity for* $L_{i}\geq 0,\;\;i=1,2,\ldots ,\;6.$

**Proof.**  Let the kernels be:

(7) $$f_{1}\left(t,\;S_{N}\right)=\Lambda -\alpha_{1}S_{N}I-\left(\theta +µ\right)S_{N},\;$$

(8) $$f_{2}\left(t,\;S_{V}\right)=\theta S_{N}-\alpha_{2}S_{V}I-\left(\phi +µ\right)S_{V},\;$$

(9) $$f_{3}\left(t,\;E\right)=\alpha_{1}S_{N}I+\alpha_{2}S_{V}I-\left(\beta +µ\right)E,\;$$

(10) $$f_{4}\left(t,\;I\right)=\beta E-\left(\eta +\gamma +d_{1}+µ\right)I,\;$$

(11) $$f_{5}\left(t,\;H\right)=\eta I-\left(\pi +d_{2}+µ\right)H,\;$$

(12) $$f_{6}\left(t,\;R\right)=\gamma I+\phi S_{V}+\pi H-µR.\;$$

Now

$$\begin{array}{c} \left|f_{1}\left(t,\;S_{N}\right)-f_{1}\left(t,\;S_{N}^{*}\right)\right|=\left|\left(\alpha_{1}I+\theta +µ\right)\left(S_{N}-S_{N}^{*}\right)\right|\\ \leq \left(\left|\theta +µ\right|+\left|\alpha_{1}I\right|\right)\|S_{N}-S_{N}^{*}\|\\ \leq (\left|\theta +µ\right|+\alpha_{1}\underset{t\in \left[0,\;h^{*}\right]}{\max}\left|I\left(t\right)\right|)\|S_{N}-S_{N}^{*}\|\\ \leq L_{1}\|S_{N}-S_{N}^{*}\|,\;L_{1}=\left|\theta +µ\right|+\alpha_{1}\underset{t\in \left[0,\;h^{*}\right]}{\max}\left|I\left(t\right)\right|. \end{array}$$

Hence,

(13) $$\left|f_{1}\left(t,\;S_{N}\right)-f_{1}\left(t,\;S_{N}^{*}\right)\right|\leq L_{1}\|S_{N}-S_{N}^{*}\|.\;$$

In a similar way, we obtain:

(14) $$\|f_{2}\left(t,\;S_{V}\right)-f_{2}\left(t,\;S_{V}^{*}\right)\|\leq L_{2}\|S_{V}-S_{V}^{*}\|,$$

(15) $$\|f_{3}\left(t,\;E\right)-f_{3}\left(t,\;E^{*}\right)\|\leq L_{3}\|E-E^{*}\|,\;$$

(16) $$\|f_{4}\left(t,\;I\right)-f_{4}\left(t,\;I^{*}\right)\|\leq L_{4}\|I-I^{*}\|,\;$$

(17) $$\|f_{5}\left(t,\;H\right)\|-f_{5}\left(t,\;H^{*}\right)\|\leq L_{5}\|H-H^{*}\|,\;$$

(18) $$\|f_{6}\left(t,\;R\right)-f_{6}\left(t,\;R^{*}\right)\|\leq L_{6}\|R-R^{*}\|.\;$$

where

$$L_{2}=\left|\phi +µ\right|+\alpha_{2}\underset{t\in \left[0,\;h^{*}\right]}{\max}\left|I\left(t\right)\right|,\;L_{3}=\left|\beta +µ\right|,\;L_{4}=\left|\eta +\gamma +d_{1}+µ\right|,\;L_{5}=\left|\pi +d_{2}+µ\right|,\;and\;L_{6}=\left|µ\right|.$$

The following lemma converts the system to Volterra-integral equations.  □

**Lemma** **1.**  *The continuous system (1) through (6) can be transformed to equivalent Volterra-integral equations.*

**Proof.**  Consider

D0CtαSNt=f1t, SNt

On integrating fractionally:

D0Ct−αD0CtαSNt=D0Ct−α[f1t, SNt]

$$S_{N}\left(t\right)-S_{N}\left(0\right)=\frac{1}{\Gamma \left(\alpha \right)}\int_{0}^{t}{\left(t-\tau \right)}^{\alpha -1}f_{1}\left(\tau ,\;S_{N}\left(\tau \right)\right)dt$$

(19) $$S_{N}\left(t\right)=S_{N}\left(0\right)+\frac{1}{\Gamma \left(\alpha \right)}\int_{0}^{t}{\left(t-\tau \right)}^{\alpha -1}f_{1}\left(\tau ,\;S_{N}\left(\tau \right)\right)dt.$$

Similarly,

(20) $$S_{V}\left(t\right)=S_{V}\left(0\right)+\frac{1}{\Gamma \left(\alpha \right)}\int_{0}^{t}{\left(t-\tau \right)}^{\alpha -1}f_{2}\left(\tau ,\;S_{V}\left(\tau \right)\right)dt,$$

(21) $$E\left(t\right)=E\left(0\right)+\frac{1}{\Gamma \left(\alpha \right)}\int_{0}^{t}{\left(t-\tau \right)}^{\alpha -1}f_{3}\left(\tau ,\;E\left(\tau \right)\right)dt,$$

(22) $$I\left(t\right)=I\left(0\right)+\frac{1}{\Gamma \left(\alpha \right)}\int_{0}^{t}{\left(t-\tau \right)}^{\alpha -1}f_{4}\left(\tau ,\;I\left(\tau \right)\right)dt,$$

(23) $$H\left(t\right)=H\left(0\right)+\frac{1}{\Gamma \left(\alpha \right)}\int_{0}^{t}{\left(t-\tau \right)}^{\alpha -1}f_{5}\left(\tau ,\;H\left(\tau \right)\right)dt,$$

(24) $$R\left(t\right)=R\left(0\right)+\frac{1}{\Gamma \left(\alpha \right)}\int_{0}^{t}{\left(t-\tau \right)}^{\alpha -1}f_{6}\left(\tau ,\;R\left(\tau \right)\right)dt.$$

 □

The following theorem gives the existence of the unique solution.

**Theorem5.**  *As in [9], let *$0<\alpha <1,\;I=\left[0,\;h^{*}\right]\subseteq \mathbb{R},\;and\;J=\left|S_{N}\left(t\right)-S_{N}\left(0\right)\right|\leq k_{1}$.

*Let*$f_{1}:I\times J\;\to \;\mathbb{R}\;$ *be a continuous bounded function, that is, * ∃!M>0 *such that* $\left|f_{i}\left(t,{\;S}_{N}\right)\right|\leq M_{1}.$

*Assume that* $f_{1}$ *satisfies Lipschitz conditions. If* $L_{1}k_{1}<M_{1}$, *then there exists unique* a $S_{V}\in C\left[0,{\;h}^{*}\right],\;where\;h^{*}=\min[h,\;\left(\frac{k_{1}\Gamma \left(\alpha +1\right)}{M_{1}}){\;}^{\frac{1}{\alpha }}\right]$, *that holds the Equation (1).*

**Proof.** 
$$Let\;T=\left\{S_{N}\in C\left[0,\;h^{*}\right]:\;\|S_{N}\left(t\right)-S_{N}\left(0\right)\|\leq k_{1}\right\}$$

Since T⊆ℝ and its closed set, then T is a complete metric space.

Recall that

(25) $$S_{N}\left(t\right)=S_{N}\left(0\right)+\frac{1}{\Gamma \left(\alpha \right)}\int_{0}^{t}{\left(t-\tau \right)}^{\alpha -1}f_{1}\left(\tau ,\;S_{N}\left(\tau \right)\right)dt$$

Define operator F in T:

(26) $$FS_{N}\left(t\right)=S_{N}\left(0\right)+\frac{1}{\Gamma \left(\alpha \right)}\int_{0}^{t}{\left(t-\tau \right)}^{\alpha -1}f_{1}\left(\tau ,\;S_{N}\left(\tau \right)\right)dt$$

To show that (26) satisfies Theorem 1, we have:

$$\begin{array}{c} \left|FS_{N}\left(t\right)-S_{N}\left(0\right)\right|=\left|\frac{1}{\Gamma \left(\alpha \right)}\int_{0}^{t}{\left(t-\tau \right)}^{\alpha -1}f_{1}\left(\tau ,\;S_{N}\left(\tau \right)\right)dt\right|\\ \leq \frac{1}{\Gamma \left(\alpha \right)}\int_{0}^{t}{\left(t-\tau \right)}^{\alpha -1}\left|f_{1}\left(\tau ,\;S_{N}\left(\tau \right)\right)\right|dt\\ \leq \frac{1}{\Gamma \left(\alpha \right)}\int_{0}^{t}{\left(t-\tau \right)}^{\alpha -1}M_{1}dt\\ =\frac{M_{1}}{\Gamma \left(\alpha +1\right)}t^{\alpha }\\ =\frac{M_{1}}{\Gamma \left(\alpha +1\right)}{\left(h^{*}\right)}^{\alpha }\\ \leq \frac{M_{1}}{\Gamma \left(\alpha +1\right)}\frac{k_{1}\Gamma \left(\alpha +1\right)}{M_{1}}\\ =k_{1} \end{array}$$

(27) $$\left|FS_{N}\left(t\right)-S_{N}\left(0\right)\right|\leq k_{1}.$$

Similarly,

(28) $$\left|FS_{V}\left(t\right)-S_{V}\left(0\right)\right|\leq k_{2},\;$$

(29) $$\left|FE\left(t\right)-E\left(0\right)\right|\leq k_{3},\;$$

(30) $$\left|FI\left(t\right)-I\left(0\right)\right|\leq k_{4},\;$$

(31) $$\left|FH\left(t\right)-H\left(0\right)\right|\leq k_{5},\;$$

(32) $$\left|FR\left(t\right)-R\left(0\right)\right|\leq k_{6}.\;$$

Therefore, F maps T onto itself.

Secondly, to show that T is contractive, we have:

$$FS_{N}-FS_{N}^{*}=S_{N}\left(0\right)-S_{N}^{*}\left(0\right)+\frac{1}{\Gamma \left(\alpha \right)}\int_{0}^{t}{\left(t-\tau \right)}^{\alpha -1}\left[f_{1}\left(\tau ,\;S_{N}\left(\tau \right)\right)-f_{1}\left(\tau ,\;S_{N}^{*}\left(\tau \right)\right)\right]d\tau .$$

Since $S_{N}\left(0\right)=S_{N}^{*}\left(0\right),$

$$\begin{array}{c} \left|FS_{N}-FS_{N}^{*}\right|=\left|\frac{1}{\Gamma \left(\alpha \right)}\int_{0}^{t}{\left(t-\tau \right)}^{\alpha -1}\left[f_{1}\left(\tau ,\;S_{N}\left(\tau \right)\right)-f_{1}\left(\tau ,\;S_{N}^{*}\left(\tau \right)\right)\right]d\tau \right|\\ \leq \frac{1}{\Gamma \left(\alpha \right)}\int_{0}^{t}{\left(t-\tau \right)}^{\alpha -1}\left|f_{1}\left(\tau ,\;S_{N}\left(\tau \right)\right)-f_{1}\left(\tau ,\;S_{N}^{*}\left(\tau \right)\right)\right|d\tau .\\ \leq \frac{1}{\Gamma \left(\alpha \right)}\int_{0}^{t}{\left(t-\tau \right)}^{\alpha -1}L_{1}\|S_{N}-S_{N}^{*}\|d\tau \\ =\frac{L_{1}}{\Gamma \left(\alpha \right)}\|S_{N}-S_{N}^{*}\|\int_{0}^{t}{\left(t-\tau \right)}^{\alpha -1}\tau^{0}d\tau \\ =\frac{L_{1}}{\Gamma \left(\alpha \right)}\|S_{N}-S_{N}^{*}\|\frac{\Gamma \left(\alpha \right)}{\Gamma \left(\alpha +1\right)}t^{\alpha }\\ =\frac{L_{1}}{\Gamma \left(\alpha +1\right)}\|S_{N}-S_{N}^{*}\|t^{\alpha }\\ \leq \frac{L_{1}}{\Gamma \left(\alpha +1\right)}\|S_{N}-S_{N}^{*}\|{\left(h^{*}\right)}^{\alpha }\\ \leq \frac{L_{1}}{\Gamma \left(\alpha +1\right)}\|S_{N}-S_{N}^{*}\|\frac{k_{1}\Gamma \left(\alpha +1\right)}{M_{1}}. \end{array}$$

Hence,

(33) $$\|FS_{N}-FS_{N}^{*}\|\leq \frac{L_{1}k_{1}}{M_{1}}\|S_{N}-S_{N}^{*}\|.\;$$

Since by hypothesis $\frac{L_{1}k_{1}}{M_{1}}<1$, then T is contractive and has a unique fixed point.

Thus, Equation (1) has a unique solution.

In a similar way, we obtained:

(34) $$\|FS_{V}-FS_{V}^{*}\|\leq \frac{L_{2}k_{2}}{M_{2}}\|S_{V}-S_{V}^{*}\|,\;$$

(35) $$\|FE-FE^{*}\|\leq \frac{L_{3}k_{3}}{M_{3}}\|E-E^{*}\|,\;$$

(36) $$\|FI-FI^{*}\|\leq \frac{L_{4}k_{4}}{M_{4}}\|I-I^{*}\|,\;$$

(37) $$\|FH-FH^{*}\|\leq \frac{L_{5}k_{5}}{M_{5}}\|H-H^{*}\|,\;$$

(38) $$\|FR-FR^{*}\|\leq \frac{L_{6}k_{6}}{M_{6}}\|R-R^{*}\|.\;$$

 □

## 5. Stability Analysis and Derivation of Basic Reproduction Ratio

In this section, we find the equilibrium solutions and conduct local stability analysis of the solutions.

Since $N=S_{N}+S_{V}+E+I+H+R,$ then for the analysis we can consider Equations (1)–(5).

### 5.1. Equilibriums Solutions and Basic Reproduction Ratio

To find the equilibrium solutions, we equate system (1) through (5) to zero:

(39) $$0=\Lambda -\alpha_{1}S_{N}I-\left(\theta +µ\right)S_{N},\;$$

(40) $$0=\theta S_{N}-\alpha_{2}S_{V}I-\left(\phi +µ\right)S_{V},\;$$

(41) $$0=\alpha_{1}S_{N}I+\alpha_{2}S_{V}I-\left(\beta +µ\right)E,\;$$

(42) $$0=\beta E-\left(\eta +\gamma +d_{1}+µ\right)I,\;$$

(43) $$0=\eta I-\left(\pi +d_{2}+µ\right)H,\;$$

Solving (39) and (40) simultaneously, we find two equilibrium solutions, disease-free and endemic equilibria.

Disease-free equilibrium $\left(E_{0}\right)$ is obtained as:

$$E_{0}=\left(S_{N}^{*},\;S_{V}^{*},\;E^{*},I^{*},H^{*}\right)=\left(\frac{\Lambda }{\theta +µ},\frac{\theta \Lambda }{\left(\theta +µ\right)\left(\phi +µ\right)},0,0,0\right).$$

The endemic equilibrium $\left(E_{1}\right)$ is obtained as:

$$E_{1}=\left(S_{N}^{**},\;S_{V}^{**},\;E^{**},I^{**},H^{**}\right).$$

Here,

(44) $$S_{N}^{**}=\frac{\Lambda }{\alpha_{1}I^{**}+\theta +\mu },\;$$

(45) $$S_{V}^{**}=\frac{\theta \Lambda }{\left(\alpha_{1}I^{**}+\theta +\mu \right)\left(\alpha_{2}I^{**}+\phi +\mu \right)},\;$$

(46) $$E^{**}=\frac{\Lambda \alpha_{1}I^{**}\left(\alpha_{1}I^{**}+\theta +\mu \right)\left(\alpha_{2}I^{**}+\phi +\mu \right)+\theta \Lambda \alpha_{2}I^{**}\left(\left(\alpha_{1}I^{**}+\theta +\mu \right)\right)}{\left(µ+\beta \right)\left(\alpha_{2}I^{**}+\phi +\mu \right){\left(\alpha_{1}I^{**}+\theta +\mu \right)}^{2}},\;$$

(47) $$\;H^{**}=\frac{\eta I^{**}}{\pi +d_{2}+µ},\;$$

and $I^{**}$ can be obtained by solving the following quadratic equations:

$$\alpha_{1}\alpha_{2}\left(µ+\beta \right)\left(\eta +\gamma +d_{1}+µ\right){\left(I^{**}\right)}^{2}+\left[\left(µ+\beta \right)\left(\eta +\gamma +d_{1}+µ\right)\left(\alpha_{1}\left(\phi +\mu \right)+\alpha_{2}\left(\theta +\mu \right)\right)-\Lambda \alpha_{1}\beta \alpha_{2}\right]I^{**}\;-\left[\Lambda \alpha_{1}\beta \left(\phi +\mu \right)+\theta \Lambda \alpha_{2}\beta \right]=0.$$

Let

$$\begin{array}{c} A=µ+\beta ,\;B=\eta +\gamma +d_{1}+µ,\;C=\phi +\mu ,D=\theta +\mu ,\;E=\Lambda \alpha_{2}\beta .\\ \alpha_{1}\alpha_{2}AB{\left(I^{**}\right)}^{2}+\left[AB\left(\alpha_{1}C+\alpha_{2}D\right)-\alpha_{1}E\right]I^{**}-E\left(C+\theta \right)=0. \end{array}$$

We have a positive root if $AB\left(\alpha_{1}C+\alpha_{2}D\right)-\alpha_{1}E<0,$ which implies:

$$\frac{\alpha_{1}E}{AB\left(\alpha_{1}C+\alpha_{2}D\right)}>1.$$

Let

$$\frac{\alpha_{1}E}{AB\left(\alpha_{1}C+\alpha_{2}D\right)}=R_{0}.$$

Hence, the basic reproduction ratio ($R_{0}$), which defines the number of secondary infections caused by a single infectious individual in a completely susceptible population, is given as:

$$R_{0}=\frac{\Lambda \alpha_{1}\beta \alpha_{2}}{\left(µ+\beta \right)\left(\eta +\gamma +d_{1}+µ\right)\left(\alpha_{1}\left(\phi +\mu \right)+\alpha_{2}\left(\theta +\mu \right)\right)}.$$

### 5.2. Local Stability Analysis of the Equilibria

From Equations (1) through (5), we construct the following Jacobian matrix:

$$J=\left[\begin{array}{ccccc} -\alpha_{1}I-\left(\theta +µ\right) & 0 & 0 & -\alpha_{1}S_{N} & 0\\ \theta & \alpha_{2}I-\left(\phi +µ\right) & 0 & -\alpha_{2}S_{V} & 0\\ \alpha_{1}I & \alpha_{2}I & -\left(\beta +µ\right) & \alpha_{1}S_{N}+\alpha_{2}S_{V} & 0\\ 0 & 0 & \beta & -\left(\eta +\gamma +d_{1}+µ\right) & 0\\ 0 & 0 & 0 & \eta & -\left(\pi +d_{2}+µ\right) \end{array}\right]$$

**Theorem** **6.**  *The disease-free equilibrium* $E_{0}$ *is locally asymptotically stable if*$R_{0}<1$.

**Proof.**  Consider the Jacobian matrix at disease-free equilibrium:

$$J\left(E_{0}\right)=\left[\begin{array}{ccccc} -\left(\theta +µ\right) & 0 & 0 & -\alpha_{1}S_{N}{}^{*} & 0\\ \theta & -\left(\phi +µ\right) & 0 & -\alpha_{2}S_{V}{}^{*} & 0\\ 0 & 0 & -\left(\beta +µ\right) & \alpha_{1}S_{N}{}^{*}+\alpha_{2}S_{V}{}^{*} & 0\\ 0 & 0 & \beta & -\left(\eta +\gamma +d_{1}+µ\right) & 0\\ 0 & 0 & 0 & \eta & -\left(\pi +d_{2}+µ\right) \end{array}\right]$$

The eigenvalues of this matrix are:

$$\lambda_{1}=-\left(\theta +µ\right)<0,\;\lambda_{2}=-\left(\phi +µ\right)<0,\;\lambda_{3}=-\left(\pi +d_{2}+µ\right)<0.$$

$\lambda_{4}\;and\;\lambda_{5}$ can be obtained by solving the following equation:

$$\begin{array}{c} -\beta \left(\alpha_{1}S_{N}{}^{*}+\alpha_{2}S_{V}{}^{*}\right)+\left(-\left(\beta +µ\right)-\lambda \right)\left(-\left(\eta +\gamma +d_{1}+µ\right)-\lambda \right)=0,\\ \lambda =\frac{-F\pm \sqrt{F^{2}-4\left[\left(\beta +µ\right)\left(\eta +\gamma +d_{1}+µ\right)-\beta \left(\alpha_{1}S_{N}{}^{*}+\alpha_{2}S_{V}{}^{*}\right)\right]}}{2}, \end{array}$$

where

$$F=\left(\eta +\gamma +d_{1}+2µ+\beta \right).$$

We can clearly see that

$$\lambda_{4}=\frac{-F-\sqrt{F^{2}-4\left[\left(\beta +µ\right)\left(\eta +\gamma +d_{1}+µ\right)-\beta \left(\alpha_{1}S_{N}{}^{*}+\alpha_{2}S_{V}{}^{*}\right)\right]}}{2}<0,$$

and

$$\lambda_{5}=\frac{-F+\sqrt{F^{2}-4\left[\left(\beta +µ\right)\left(\eta +\gamma +d_{1}+µ\right)-\beta \left(\alpha_{1}S_{N}{}^{*}+\alpha_{2}S_{V}{}^{*}\right)\right]}}{2}.$$

$\lambda_{5}<0$ if $\left(\beta +µ\right)\left(\eta +\gamma +d_{1}+µ\right)-\beta \left(\alpha_{1}S_{N}{}^{*}+\alpha_{2}S_{V}{}^{*}\right)>0.$

This implies that

$$\frac{\beta \left(\alpha_{1}S_{N}{}^{*}+\alpha_{2}S_{V}{}^{*}\right)}{\left(\beta +µ\right)\left(\eta +\gamma +d_{1}+µ\right)}<1.$$

Substituting the values of $S_{N}{}^{*}$ and $S_{V}{}^{*},$ we can clearly see that $\lambda_{5}<0\;\;\mathrm{if}\;\;R_{0}<1.$  □

**Theorem7.**  *The endemic equilibrium*$E_{1}$ *is locally asymptotically stable if*$R_{0}>1$.

**Proof.**  Consider the Jacobian matrix at $E_{1}$, we obtain:

$$J\left(E_{1}\right)=\left[\begin{array}{ccccc} -\alpha_{1}I^{*}-\left(\theta +µ\right) & 0 & 0 & -\alpha_{1}S_{N}{}^{*} & 0\\ \theta & \alpha_{2}I^{*}-\left(\phi +µ\right) & 0 & -\alpha_{2}S_{V}{}^{*} & 0\\ \alpha_{1}I^{*} & \alpha_{2}I^{*} & -\left(\beta +µ\right) & \alpha_{1}S_{N}{}^{*}+\alpha_{2}S_{V}{}^{*} & 0\\ 0 & 0 & \beta & -\left(\eta +\gamma +d_{1}+µ\right) & 0\\ 0 & 0 & 0 & \eta & -\left(\pi +d_{2}+µ\right) \end{array}\right].$$

The eigenvalues of these matrix are: $\lambda_{1}=-\left(\pi +d_{2}+µ\right)<0,$ and the remaining eigenvalues can be obtained from the following equation.

$$\begin{array}{l} \lambda^{4}+\left[\alpha_{1}I^{*}+\left(\theta µ\right)+\alpha_{2}I^{*}+\left(\phi +µ\right)+\left(\beta +µ\right)+\left(\eta +\gamma +d_{1}+µ\right)\right]\lambda^{3}\\ \;\;\;\;\;\;+[\left(\alpha_{1}I^{*}+\left(\theta +µ\right)\right)\left(\alpha_{2}I^{*}+\left(\phi +µ\right)\right)\\ \;\;\;\;\;\;+\left(\alpha_{1}I^{*}+\left(\theta +µ\right)+\alpha_{2}I^{*}+\left(\phi +µ\right)\right)\left(\left(\beta +µ\right)+\left(\eta +\gamma +d_{1}+µ\right)\right)\\ \;\;\;\;\;\;+\left(\beta +µ\right)\left(\eta +\gamma +d_{1}+µ\right)-{\left(\alpha_{1}S_{N}{}^{*}+\alpha_{2}S_{V}{}^{*}\right)}^{2}]\lambda^{2}\\ \;\;\;\;\;\;+[\left(\alpha_{1}I^{*}+\left(\theta +µ\right)\right)\left(\alpha_{2}I^{*}+\left(\phi +µ\right)\right)\left(\left(\beta +µ\right)+\left(\eta +\gamma +d_{1}+µ\right)\right)\\ \;\;\;\;\;\;+\left(\left(\beta +µ\right)+\left(\eta +\gamma +d_{1}+µ\right)\right)\left(\left(\alpha_{1}I^{*}+\left(\theta +µ\right)\right)\left(\alpha_{2}I^{*}+\left(\phi +µ\right)\right)\right)\\ \;\;\;\;\;\;+\left(\alpha_{1}S_{N}{}^{*}+\alpha_{2}S_{V}{}^{*}\right)\alpha_{2}S_{V}{}^{*}\alpha_{1}I^{*}-{\left(\alpha_{1}S_{N}{}^{*}+\alpha_{2}S_{V}{}^{*}\right)}^{2}(\alpha_{1}I^{*}+\left(\theta +µ\right)\\ \;\;\;\;\;\;+\alpha_{2}I^{*}+\left(\phi +µ\right))]\lambda \\ \;\;\;\;\;\;+[\left(\alpha_{1}I^{*}+\left(\theta +µ\right)\right)\left(\alpha_{2}I^{*}+\left(\phi +µ\right)\right)\left(\beta +µ\right)+\left(\eta +\gamma +d_{1}+µ\right)\\ \;\;\;\;\;\;+\alpha_{1}S_{N}{}^{*}\left(\alpha_{1}S_{N}{}^{*}+\alpha_{2}S_{V}{}^{*}\right)\left(\theta \alpha_{2}I^{*}+\alpha_{1}I^{*}\left(\alpha_{2}I^{*}+\left(\phi +µ\right)\right)\right)\\ \;\;\;\;\;\;+\left(\alpha_{1}S_{N}{}^{*}+\alpha_{2}S_{V}{}^{*}\right)\left(\alpha_{1}I^{*}+\left(\theta +µ\right)\right)\alpha_{2}I^{*}\alpha_{2}S_{V}{}^{*}\\ \;\;\;\;\;\;-{\left(\alpha_{1}S_{N}{}^{*}+\alpha_{2}S_{V}{}^{*}\right)}^{2}\left(\alpha_{1}I^{*}+\left(\theta +µ\right)\right)\left(\alpha_{2}I^{*}+\left(\phi +µ\right)\right)]=0. \end{array}$$

By the Routh–Hurwitz stability criterion, the remaining eigenvalues are negative if:

$$a_{4}>0,\;a_{3}>0,\;a_{1}>0,\;and\;a_{1}a_{2}a_{3}-a_{3}{}^{2}-a_{4}a_{1}{}^{2}>0.$$

Now,

$$a_{4}=1>0,\;a_{3}=\alpha_{1}I^{*}+\left(\theta +µ\right)+\alpha_{2}I^{*}+\left(\phi +µ\right)+\left(\beta +µ\right)+\left(\eta +\gamma +d_{1}+µ\right)>0,$$

$$\begin{array}{l} a_{1}=[\left(\alpha_{1}I^{*}+\left(\theta +µ\right)\right)\left(\alpha_{2}I^{*}+\left(\phi +µ\right)\right)\left(\beta +µ\right)+\left(\eta +\gamma +d_{1}+µ\right)\\ \;\;\;\;+\alpha_{1}S_{N}{}^{*}\left(\alpha_{1}S_{N}{}^{*}+\alpha_{2}S_{V}{}^{*}\right)\left(\theta \alpha_{2}I^{*}+\alpha_{1}I^{*}\left(\alpha_{2}I^{*}+\left(\phi +µ\right)\right)\right)\\ \;\;\;\;+\left(\alpha_{1}S_{N}{}^{*}+\alpha_{2}S_{V}{}^{*}\right)\left(\alpha_{1}I^{*}+\left(\theta +µ\right)\right)\alpha_{2}I^{*}\alpha_{2}S_{V}{}^{*}-{\left(\alpha_{1}S_{N}{}^{*}+\alpha_{2}S_{V}{}^{*}\right)}^{2}\left(\alpha_{1}I^{*}+\left(\theta +µ\right)\right)\left(\alpha_{2}I^{*}+\left(\phi +µ\right)\right)]. \end{array}$$

It can easily be seen that $a_{1}>0,\;$ and $a_{1}a_{2}a_{3}-a_{3}{}^{2}-a_{4}a_{1}{}^{2}>0,$ if $R_{0}>1.$  □

## 6. Numerical Simulations and Data Fitting

To illustrate the theoretical results, numerical simulations are performed.For the numerical simulations, we use the following parameter values from [27]: $\Lambda =130,\;\alpha_{1}=0.0011,\alpha_{2}=0.00011,\theta =0.25,\mu =0.0395,\;\beta =0.0689,\;\phi =0.02,\;\eta =0.009,\gamma =0.098,\;\pi =0.0714,\;d_{1}=0.015,\;d_{2}=0.015,\alpha \in \left.(0,1\right]$. Using the Caputo fractional derivative of order and implicit Euler’s approximation discussed in [28,29], numerical simulations of the resulting systems are performed.

**Figure 2:**
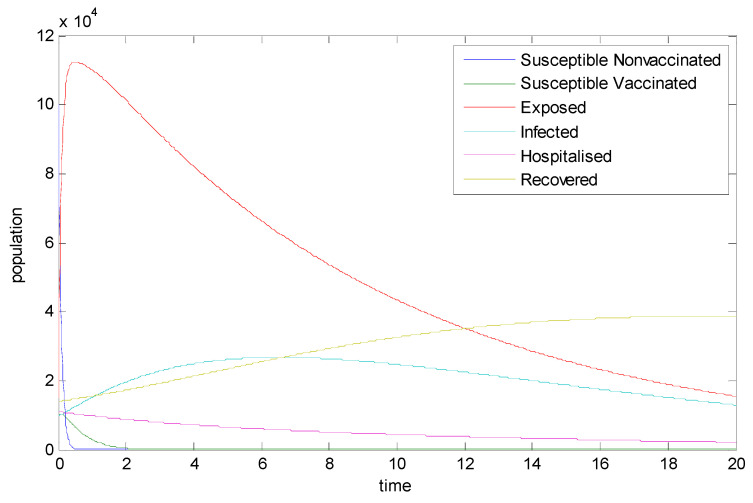
Numerical simulations of the system of FODE (1)–(6).

Figure 2 depicts the dynamics of the model. It can be clearly seen that the susceptible populations all go to extinction, whereas the infected, exposed, and hospitalized populations proliferate. This clearly shows the need for increasing the vaccination level.

**Figure 3:**
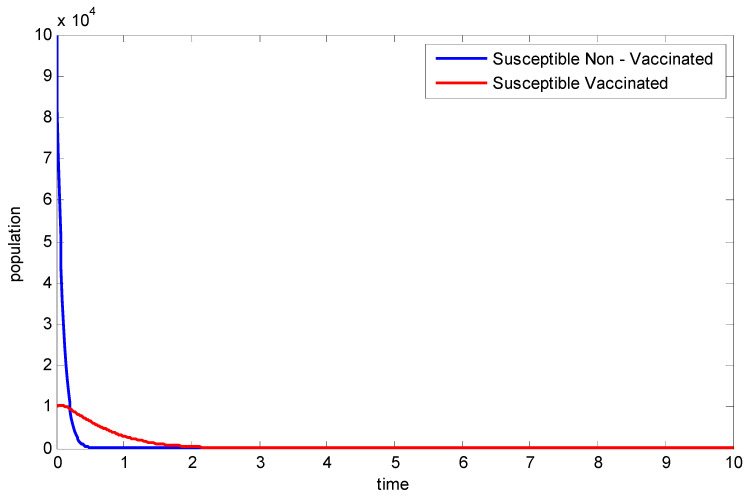
Comparing the vaccinated and non-vaccinated susceptible populations.

From Figure 3, it can be observed that the population of the non-vaccinated individuals goes to extinction faster than that of the vaccinated people. Hence, the significance of vaccination.

**Figure 4:**
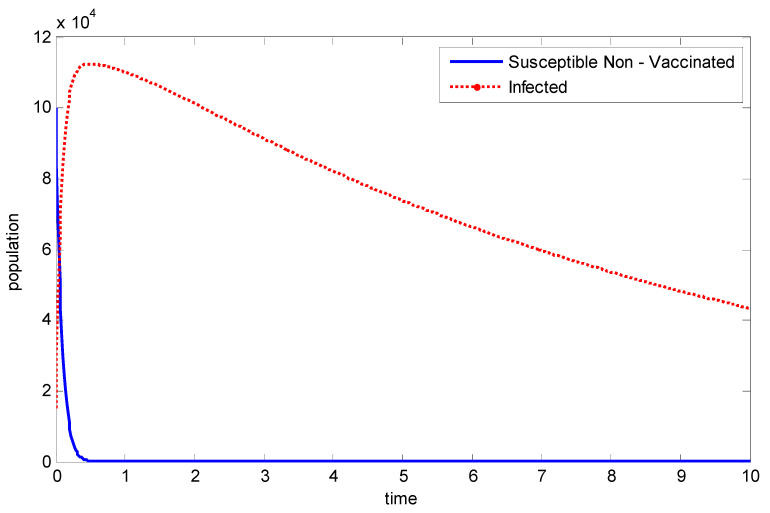
Comparing the susceptible non-vaccinated population with the exposed population.

Figure 4 compares the population of exposed individuals and non-vaccinated individuals. It can be clearly observed that immediately the epidemic starts, and without exercising vaccination, the population of exposed individuals then explodes.

**Figure 5:**
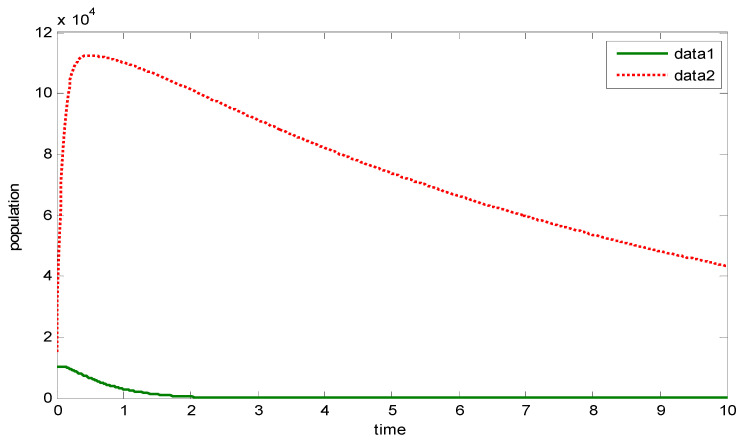
Comparing the susceptible vaccinated population with the exposed population.

Figure 5 compares the population of exposed individuals and vaccinated individuals. It can be clearly observed that although the population of vaccinated individuals goes to extinction, it takes time for this to occur compared with Figure 4. Hence, there is a need for an increase in the level of vaccination coverage.

**Figure 6:**
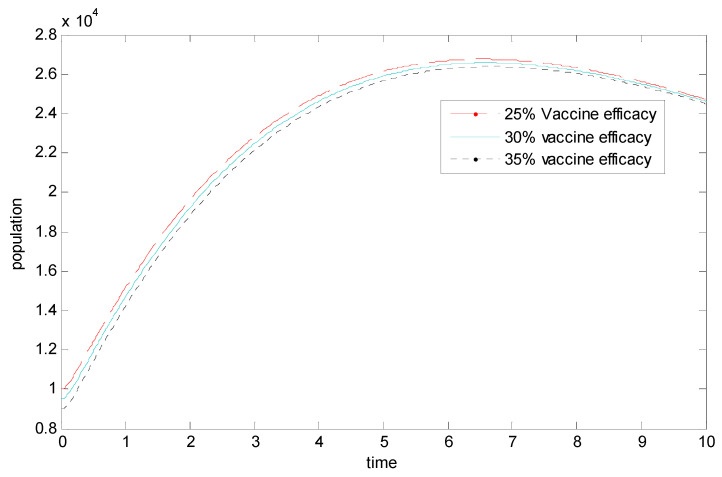
Comparing the vaccine efficacy in the infected population.

Figure 6 compares the vaccine efficacy in the infected population. It can be clearly seen that increasing the efficacy leads to a decrease in the population of infected individuals. Hence the need for vaccination in controlling the spread of the disease.

**Figure 7:**
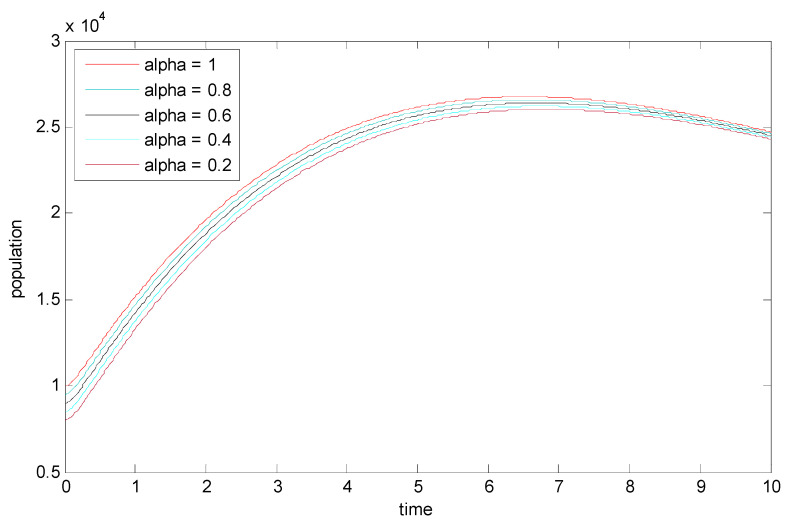
Dynamics of the infected population for various values of α.

Figure 7 shows the influence of the variation in the fractional-order α on the biological behavior of the infected population. It is clear from this figure that the population has a decreasing effect when α is decreased from 0.2 to 1. Hence, the memory effect can be clearly seen.

### Model Fitting

To fit the data, we used confirmed COVID-19 case data from 12 January through 12 May 2020. The Department of Disease Control, Thailand Ministry of Public Health (DDC), provides this publicly available, daily updated data at (https://data.go.th/dataset/covid-19-daily, accessed on 27 November 2022). Following the approach described in [30], the underlying model system is fitted with the given data.

**Figure 8:**
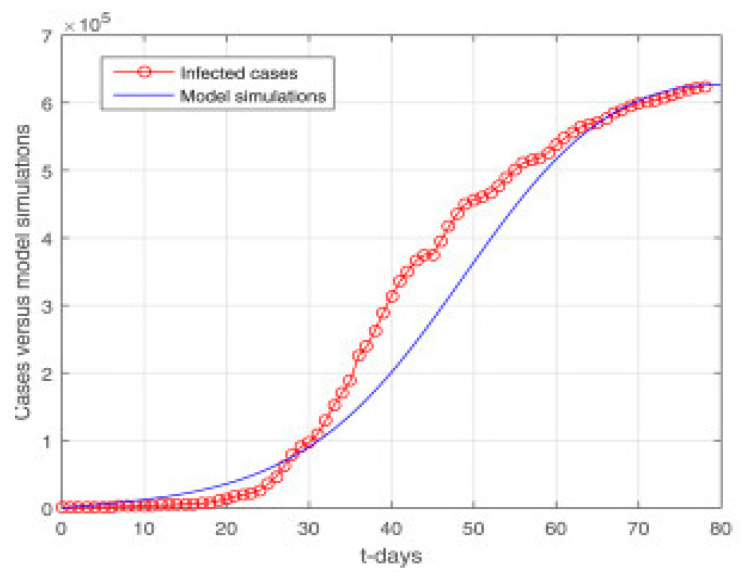
Model fitting using real data.

**Figure 9:**
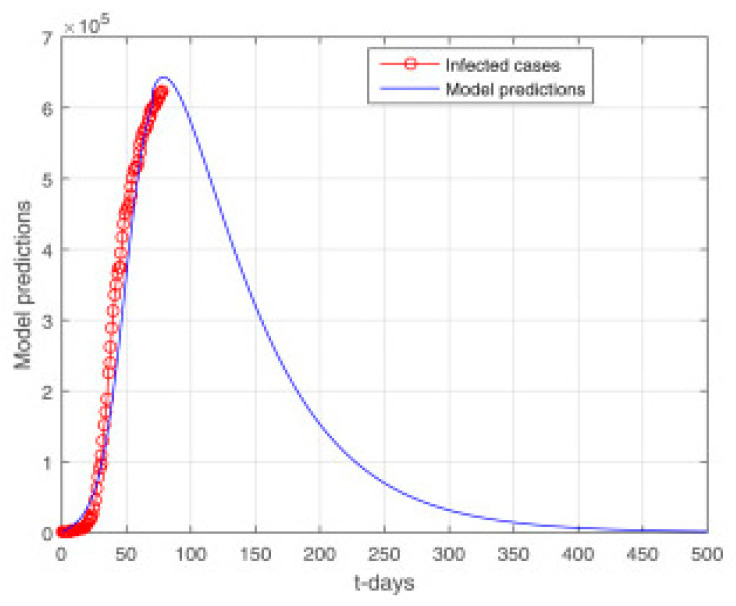
Model prediction using real data.

Some cases are reviewed on a daily basis. Using the nonlinear least squares method, we fitted our model to the available data. To obtain the best fit of the model, we repeated the experiments multiple times. Nonlinear least squares curve fitting provides realistic parameter values. Figure 8 shows the model fitting using real data. In Figure 9, the long-term predictions of the model can be seen. In order for the simple model to approximate the complex one, the susceptible class S must have the same number of individuals in both models (when comparing compartment-wise). MatLab’s optimization function create Optim Problem was used to fit the observed data.

In order to inform policy decisions about which parameters to focus on for data collection or to mitigate disease spread, early identification of model parameters with greater influence on disease transmission is important.

## 7. Discussion and Conclusions

In this study, a fractional-order model was used to model the transmission dynamics of COVID-19 with vaccine impact. The model has been validated by fitting it to four months of real COVID-19 infection data in Thailand. Predictions for a longer period are provided by the model, which provides a good fit for the data. The study examined the importance of vaccines in controlling epidemic diseases using a fractional-order mathematical model in the Caputo sense. The Banach contraction mapping principle is used to prove the existence and uniqueness of the model’s solution. In the model, asymptotic equilibrium indices for disease-free and endemic equilibrium were found and demonstrated to be locally asymptotically stable with $R_{0}<1\;$ and $\;R_{0}>1\;$, respectively. Our numerical simulations clearly demonstrate the importance of the vaccine. A comparison of vaccine efficacy in infected populations has been conducted. An increase in efficacy leads to a decrease in the number of infected individuals. Thus, vaccination is necessary to control the spread of the disease. Our results show the changes that occur at every time instant due to the variation of α. A fractional-order differential equation is a generalization of an integer-order differential equation. The use of fractional-order differential equations can help reduce the errors caused by neglected parameters in biological systems.

The data fitting and forecasting of the model for a longer period have been evaluated using real values of COVID-19 infections in Thailand over a four-month period.

The model developed in this paper is robust and can be applied to other countries’ realities. Moreover, mathematical models of this nature will shed light on some important aspects of the pandemic. Furthermore, fractional-order differential equation models of COVID-19 transmission have several advantages over classical integer-order mathematical models, which ignore the effects of memory or long-range interactions.

Future studies could investigate the impact of both therapeutic and (adherence to) nontherapeutic measures on COVID-19 dynamics. Including control variables in the model is desirable to determine the best strategy of treatment, control, and elimination of the infection, which will also be considered in future work.

## Data Availability

The data are available on request.
